# Review of Contributions to the Russian Child Well-Being Index: Focus on Subjective Well-Being Indicators

**DOI:** 10.11621/pir.2021.0313

**Published:** 2021-09-30

**Authors:** Tatyana O. Archakova, Elvira Garifulina

**Affiliations:** Victoria Charity Child Foundation, Moscow, Russia

**Keywords:** Children’s subjective well-being, childhood in Russia, national Index of Child Well-Being (CWBI)

## Abstract

**Background:**

In Russia, there is a demand for evaluation of children’s well-being, including subjective well-being, at the national and regional levels. To implement such an evaluation system, it is necessary to develop a Russian Child Well-Being Index (CWBI), which includes indicators of both objective and subjective wellbeing in several domains. One can rely on various national data sources that can be partially integrated into the CWBI, as well as the application of the UNICEF/ Innocenti methodology for children’s well-being evaluation and new developments by Russian research teams.

**Objective:**

To analyze the Russian experience in developing approaches to large-scale and multidimensional evaluation of children’s well-being (with an emphasis on subjective well-being) and to provide recommendations for development of the national Children’s Well-Being Index (CWBI).

**Design:**

Scoping review of the methodology and results of the studies that can inform the Russian task force on CWBI development.

**Results:**

Like most international models of subjective well-being, a Russian CWBI will be based on various aspects of the socio-ecological approach. The structure of domains vary but is generally compatible with the UNICEF/Innocenti model. The tools used by Russian researchers have included standardized psychometric techniques (as an independent tool and as a control of various types of validity); questionnaires specially designed to operationalize certain domains of well-being; and qualitative methods applied to small samples of children, such as focus groups, and creative and play-based methods. Work on the development of the CWBI (including the subjective well-being indicators) has been most actively performed in relation to children in state care; therefore, many of the tools have been designed to address the particular characteristics of this target group.

**Conclusion:**

Recommendations for development of the national Children’s Well-Being Index (CWBI) are given, including both the index design and organizational/ethical considerations.

## Introduction

One of the most important criteria for a country’s social progress is the level of its children’s well-being. The first steps toward the purposeful and informed development of the conditions required to increase children’s well-being should include reaching a consensus on defining and measuring this construct among the scientific and expert communities, governmental bodies, and wider society. The index should also take into account the views of the children themselves, including children from underrepresented and high-risk groups.

The core aspects of the concept of well-being are multidimensionality (manifestations in various fields of life) and normative character (reliance on the ideas of “good” and “bad”) (Sandin, 2013). National and international (UNICEF, OECD) indexes of children’s well-being — systems of subjective and objective indicators in several fields of life (domains) — are used to measure children’s well-being at the macrolevel.

There is increasing demand for a national CWBI in Russia. The plan for general action in the framework of the “Childhood Decade” national project (2018 to 2027) includes “performing research, including international, to evaluate the level of wellbeing, including subjective well-being, in children and adolescents.” A CWBI which allows the nation “to evaluate quality of children’s life in the Russian Federation regions” and “perform comparative analysis of child well-being levels in Russia and the other countries of the UN Convention of the Rights of the Child,” is scheduled to be designed by 2024.

The Russian expert community collected data according to several indicators of the UNICEF/Innocenti child well-being index in 2007, but the final index was not calculated for Russia because of lack of data in three domains — “Material well-being”, “Education”, and “Behaviors and risks”. The methodology of the UNICEF child well-being index was also used in preparing the report “Children in adversity: Prevention of ill-being” (Children support foundation …, 2013). Finally, Russia has participated in the OECD’s Programme for International Student Assessment (PISA), that has included study of the well-being domain since 2016 (OECD, 2016).

Currently the ministries responsible for child-related issues collect large sets of data on the objective well-being of the whole child population, as well as of the most vulnerable groups (children with disabilities, children in state care, etc.), in various domains such as observance of rights and access to social services, health, education. The part of this information which is relevant for developing the CWBI may thus be relatively easy to obtain through analysis of the data collected for the State Report on the Situation with Children and Families and the children’s ombudsman’s yearly report. Information on Russian citizens’ subjective well-being is collected within the national and departmental sets of data that are not specifically aimed at the child population, but may be analyzed through the lens of children’s well-being (*e.g.,* the Federal State Statistics Service (Rosstat) on Sustainable Development Goals).

However, one cannot make comprehensive interpretations and conclusions based solely on those objective indicators. The levels of objective well-being (*e.g.,* material assets and resources available) and subjective well-being (*e.g.*, personal satisfaction with one’s situation) may mismatch (see, for example, Heukamp & Arino, 2011). Therefore, a comprehensive well-being index should combine objective and subjective indicators in each domain, capturing both positive and negative aspects in children’s lives (Lee, 2014).

Subjective well-being is frequently defined as “the set of perceptions, evaluations and aspirations of people (in this case, children) about their own lives and living conditions” (Campbell, Converse, & Rogers, 1976). Subjective well-being of children is regarded as a construct that differs from subjective well-being of adults in its determinants: children face age-dependent developmental tasks and challenges, and their well-being is based on a balance between current “well-being” and “well-becoming,” *i.e.,* factors that ensure well-being in later life (Ben-Arieh, 2006). It is also necessary to consider well-being in typically “childish” areas of life, such as play or learning activities. When discussing well-being in children, it is important to consider two types of indicators: both the outcomes (the level of functioning of individual children) and the contexts (environment, resources, and/or inputs) needed to support that development (Moore & Theokas, 2008).

Asking children for personal opinions and emotional evaluations about their life conditions can produce new and “unexpected” knowledge (Casas, 2011). The so-called “child indicators movement” is closely connected with the “new sociology of childhood” that regards children as competent social actors with subjective opinions and assessments that are no less significant than those of their parents and educators (Ben-Arieh, 2008). Triangulating the data obtained from all three types of stakeholders — the child welfare system (objective indicators), adults, and the children themselves (subjective indicators) — is a good practice in both social research and evaluation of programs and policies. Considering children and youth to be competent actors also requires involving them in the development and approbation of CWBIs, as well as in creating measuring instruments, collecting data, and preparing reports.

An interesting experiment in collaboration with children is represented by the development of some regional subjective well-being indexes, such as the Irish Development of National Set of Child Well-Being Indicators and the Scottish Wellbeing Wheel and Wellbeing Indicators. The Russian professional community has also initiated attempts to involve children in discussing well-being and its evaluation; one example was at the “Together with Children” international conference in 2020 (see Archakova & Garifulina, 2020 for the comprehensive report).

The aim of our work was to analyze the Russian experience in developing approaches to large-scale and multidimensional evaluation of children’s well-being (with an emphasis on the measurement of subjective well-being in each domain), and to make recommendations for the development of the national Children’s Well-Being Index (CWBI).

## Method

This article provides a scoping review (Munn et al., 2018) of publications that were selected by the Russian CWBI task force and seeks to aid the taskforce’s work with their methodology and results.

The scoping review included research that: 1) focused on measuring children’s well-being in several domains and provided a framework for understanding their structure; 2) used subjective well-being indicators; and 3) was performed on large (regional/Russian-wide) samples (for quantitative research) or referred to the opinions and assessments of the children themselves (for desk studies and focus groups).

We excluded research on children’s subjective well-being that measured wellbeing in only one domain, *e.g.,* dealing with subjective well-being in primary care and the school environment. While such papers provide valuable insights for CWBI development, our review was focused on the structural characteristics of the index.

The review included six papers (see *Table 1* for their characteristics). It provided an analysis of approaches to working definitions of subjective child well-being, the construction of its domains, the measurement instruments, and issues of their validity, as well as the most significant results of their implementation.

**Table 1:** Review sources

	Population	Russian region(s)	Sample size and available characteristics	Settings	Ethical considerations	Primary disciplinary context	Type of publication
Bruk, 2019; Rees, Savahl, Lee, & Casas, 2020	General child population, 10–12 y.o.	Tyumen region	1959 (989 F), 10 y.o. — 953, 12 y.o. — 951, 82% urban, 3,7% living outside family settings	Computer testing	Guidance on key ethical principles formulated for the project; ethical approval from an appro- priate authorising body within their country	Multidisci- plinary	Research pa- per (report)
Oslon, Semya, Prokopeva & Kolesnikova, 2020	Children in state care (group homes)	85 regions	498 (248 F), mean age — 15.1 y.o. (SD — 1.34), w/o intellectual disabilities	Computer testing	Helsinki declaration	Psychology	Research paper (peer- reviewed)
Odinokova, Rusakova & Usacheva, 2017	Children in state care (group homes)	St. Petersburg (215 children), Ekatrinburg (124), Bashkorto- stan republic (178)	517 (46.3% F), 10–17 y.o., mean age — 14 y.o., normal or delayed intel- lectual development (w/o intellectual disabilities)	Personal inter- views, pen and- paper question- naires	Guidance on key ethical principles formulated for the project, reference to ethical principles of child participation in social projects evaluation	Sociology	Research paper (peer- reviewed)
Oslon, 2019	Children in state care and alumni (group homes)	64 regions	1932 children in care 4–18 y.o. (3 sub-groups: preschoolers, children 7–12 y.o., adolescents 13–18 y.o.)	Computer test- ing	N/A	Psychology	Research pa- per (confer- ence report)
Rogozin & Ipatova, 2019	Selected groups of children and birth / foster parents of such children	Voronezh (city); Ulyanovsk region	8 (4 F) children from 2-parent families; 5 (4 F) children with disabilities; 7 (6 F) children in care	Focus-groups	N/A	Sociology	Research pa- per (report)
Archakova & Garifulina, 2020	Children receiv- ing services from organiza- tions — grantees of Timchenko Foundation	30 regions	Small-scale social projects evaluations (of 15592 children participated in the projects)	Desk analysis of evaluation in- struments (in- terviews, focus- groups, narrative and play-based metods)	N/A	Sociology /social project management	Review paper (peer-re- viewed)

*Note. F = female, y.o. = years old, N/A = not available*

## Results

### Working Definition

*The Children’s Worlds: The International Survey of Children’s Well-Being*, Wave 3, 2016–2019 (ISCWeB), which dealt with the Russian child population in the Tyumen region, focused on “children’s day-to-day feelings of happiness and sadness; their satisfaction with their life as a whole and different aspects of it; their feelings of safety, being cared for, autonomy, and being listened to; and their hopes and expectations for the future” (Children’s Worlds, 2020). It reflected the importance of using plain language, as was also emphasized by Rogozin, Ipatova, & Garifulina (2019), who held focus-groups to discuss well-being and its measurement with adolescents. If plain language is not used, a discussion is rapidly “colonized” with bureaucratic ones, and alienation emerges. Rogozin & Ipatova (2019) also noted that both adolescents and parents are more familiar with defining well-being as the absence of manifestations of adversity than with any positive indicators.

Two large-scale research projects on the well-being of children in state care (children’s homes) approached the definition of well-being from different positions. Odinokova, Rusakova, and Usacheva (2018) defined subjective well-being as “the situation when a child is free from any symptoms of mental and somatic disorders; has capacities and skills which make him/her proud; is satisfied with the school environment; relationships with friends and significant adults; has at least one adult (s)he can reach for support; feels safe at the children's home and at school; is satisfied with living conditions in the children's home; and knows his/her rights and participates in decision-making.” That approach corresponds to the UNICEF/Innocenti universal three levels (individual, relationships, and context) model of well-being (Lippman, Moore, & Mcintosh, 2011).

Oslon, Semya, Prokopeva, and Kolesnikova (2020) emphasized the importance of elaborating their own working definition of well-being and making it applicable specifically to children in state care. They relied on Myasischev’s theory of attitudes and considered a child’s subjective well-being as his/her satisfaction with *the system* of his/her attitudes toward:

- him/herself (e*.g.,* positive self-esteem, satisfaction with one’s skills and achievements);
- others (*e.g.,* availability of a close adult, good relationships with peers);
- the environment (*e.g.*, physically and psychologically safe living conditions, normalization of life, knowledge of one’s rights, adults taking one’s opinion into account); and
- the chronotope (personal history, current situation and future prospects).

In the 2020 review by Archakova and Garifulina, subjective well-being was considered in the framework of monitoring and evaluating social practices (projects) and their results. Each practice provided its own working definition of well-being grounded in its design (theory of change, set of activities, logic model, and results chain) and in particular, characteristics of a target child population (*e.g.*, age, absence/presence of developmental disabilities). The results of separate practice-oriented measurements of children’s well-being were further consolidated into high-level umbrella indicators (comparable to domains).

While the reviewed definitions of well-being varied in scope and structure, it was possible to combine the insights into a general “formula:” *“Children’s well-being is [a vision of good life situation = objective factors], experienced by children as [a vision of positive cognitive and emotional assessment = subjective factors] in the presence of supportive interpersonal relationships [the most significant factor of child development, mediating his/her attitudes towards the situation].*” After being formulated, such a definition should be translated into a plain language wording.

### Domains

The works included in this scoping review had moderate variations in determining separate domains of child well-being. We attempted to compare them and highlight equivalent or synonymic ones (*Table 2*). Interestingly, the results of focus groups where children discussed the meaning of “well-being” and “happiness” (Rogozin, Ipatova, & Garifulina, 2019) supported the Innocenti/UNICEF model of well-being domains (Adamson, 2007).

**Table 2:** Child well-being domains

Oslon (2018); Oslon, Semya, Prokopeva & Kolesnikova (2020)	Odinokova, Rusakova & Usacheva (2018)	Bruk, 2019	Rogozin, Ipatova & Garifulina, 2019	Archakova & Garifulina, 2020
Skills and abilities	Education		Education	Knowledge and skills
Self-esteem & self- evaluation		Self		
Overall well-being		Overall well- being	Overall subjective well-being	Overall subjective well-being
	Mood and emo- tional states / Physical activity			Emotional wellbeing
Supportive network	Relationships with adults and peers	Family / Friends / School / Neighbourhood	Relationships with family and peers	Child-parent relationships Social communication skills
Safety	Safety		Behavior, risks and experiences of abuse / health and safety	Physical wellbeing
Rights	Knowledge of rights	Children’s rights		
Taking a child’s view into consideration	Participation in decision-making			
Normalization of life				
Satisfaction with life in general and living conditions in a chil- dren’s home	Living condi- tions in children’s homes	Home context Economic / ma- terial context	Material wellbeing	
Satisfaction with personal chronotope (past, present, future)		Time use		

**Table 3:** Instruments used in quantitative research

	Admission format	Number and types of questions	Standardized measures	Child-friendly adaptations
Bruk, 2019; Rees, Savahl, Lee, & Casas, 2020	Both paper-and-pencil and online forms are available	Total of 69 questions (set for 12 y.o.) and 80 questions (set for 10 y.o.). Close-ended questions and scales— agreement, frequency and satisfaction	Context-free: The Student Life Satisfaction Scale (SLSS, Huebner, 1991) and the one item Overall Life Satisfaction (OLS). Domain-specific: the Personal Well-being Index— School Children (PWI-SC, Cummins & Lau, 2005); The Brief Multidimensional Student Life Satisfaction Scale (BMSLSS, Seligson, Huebner & Valois, 2003); Russell’s Core Affect scale (Russell, 2003)	Consultations on correct and culture-sensitive translation and adaptation of items; child-friendly language with short and clear wordings
Oslon, 2019; Oslon, Semya, Prokopeva & Kolesnikova, 2020	Online from with technical assistance of an adult (typically, a psychologist)	10 sets of measures, including Likert-scale questionnaires, close-ended questions, sociometric visual maps	Domain-specific: Children’s Depression Inventory (Kovac, 1992); Self-Assessment Ladder (Schur, 1982); Rosenberg self-esteem scale (Rosenberg, 1965); brief version of Hardiness test (Osin & Rasskazova, 2013)	Likert-type scales using smiles The procedure is divided into several meetings to keep up with a child’s motivation and attention span
Odinokova, Rusakova & Usacheva, 2017	In-person interviews by independent interviewers. primary school children and\or children with learning disabilities; small groups interviews for adolescents	Total of 61 questions, mostly Likert-type scales, or close-ended questions (excepting the questions “Which children’s rights do you know?” and “What are you proud of?”)	–	Materials with illustrations; questions on frequency use visual scales with color gradient; Likert-type scales using smiles (based on the pediatric pain scale). Consultations with children on child-friendly language. Certificates of participation to recognize a child’s efforts

### Instruments and Validity Issues

All the research we considered used large sets of close-ended questions and scales, and all the research teams adapted them to meet children’s needs in terms of design (language, visual prompts) and procedure (timing, adult assistance).

Comparison of measurements used in two large-scale Russian research projects revealed variations by academic discipline: the “psychological” approach by Oslon et al. (2019, 2020) led to inclusion of clinical diagnostic items, such as the Children’s Depression Inventory, in contrast with the “sociological” approach by Odinokova et al. (2017), that used a standardized measure as a validity test but not as part of the questions set.

In the Children’s World project, extensive testing and statistical work was done to ensure good functioning of the items , and to check the relevance of the domains and the items for children in different socio-cultural regions. The questionnaires were comprehensively piloted in various countries and languages, using large-scale samples as well as focus groups and interviews with children (Casas & Rees, 2015).

Odinokova et al. (2018) used the Strengths and Difficulties Questionnaire to evaluate the convergent validity of their child well-being questionnaire. They also analyzed the frequency of missing answers and their correlation with well-being level using Pearson’s χ2. The most frequently skipped questions were about contacts with birth family and relatives (8.7% of answers missing), children’s rights (7%) and abuse (4%). That result significantly (p < 0.05) correlated with difficulties in interactions with peers and adults at the children’s home and psychological abuse from adults.

Oslon et al. (2020) also evaluated the convergent validity of their index scales, comparing them with standardized measures using Pearson’s χ2 and Cronbach’s alpha. The standardized measures included Rosenberg’s self-esteem scale (Rosenberg, 1965) and a short version of the Hardiness test (Osin & Rasskazova, 2013).

The qualitative research by Rogozin and Istomina (2019) employed triangulation of child participants’, parents’, and practitioners’ points of view to support content validity. Triangulation of data obtained from various groups of respondents and/or with various measures is typically implemented for monitoring and evaluating of child well-being in social projects (Archakova & Garifulina, 2020).

### Findings

The overall results of the Children’s Worlds study for the Russian (Tyumen region) sample were quite optimistic: 90.6% of the 10-year-olds and 12-year-olds were satisfied with their lives in general. A total of 78.4% of 12-year-olds felt positive about their future (Bruk, 2019). At the same time Russia ranked 26th of 35 countries in the study, basing on mean scores on life satisfaction and feelings of happiness and sadness (Children’s Worlds, 2020).

The population of children in state care, by contrast, started to worry about their futures from the age of seven. The prevalence of such worries slightly decreased in adolescence but increased again on the eve of graduation. The absolute majority of children at children’s homes had not developed an image of a preferred future (Oslon, 2018), and they strongly doubted the possibility of achieving any positive results. They did not believe they would be able to receive a good education (57%), become a successful professional (44%), or find a desirable job and earn enough (40%). A total of 39% of adolescents assessed their readiness for future independent life as insufficient (Semya, 2020).

In the Children’s Worlds study in the Russian sample, the majority of children reported themselves to be very satisfied with the people they lived with (89.2%). The most frequently reported issue was that parents did not always listen to their children and take their views into account. Satisfaction with family relationships has the greatest impact on overall life satisfaction, being the most significant predictor of SWB and overall life satisfaction in 12-years-olds (Bruk, 2019).

The most problematic field of interpersonal relationships for the Russian sample was school. Children in the 10-year-old and 12-year-old age groups rated satisfaction with family and friends higher than satisfaction with school. Just slightly over 50% of the children (totally) agreed that their teachers cared about them, listened to them, and took their views into account. A total of 32.1% of the children said that there were a lot of arguments between children in their class; 37.6% reported having been hit by schoolmates at least once in the last month; 54.6% said they had been called names; and 44.1% said they were ostracized by peers at least once during the previous month.

Focus-groups with children (Rogozin & Ipatova, 2019) have also demonstrated the high importance of family and satisfaction with family relationships for their well-being; the group of children with disabilities also highlighted the issues of social contacts and friendship with peers. In the sample of children in state care, the availability of close relationships with an adult (positive answers to the question “Do you have an adult who you can trust in your children’s home?”) significantly correlated (p<0.001) with the overall well-being scale (Odinokova, 2018). At the same time, children in state care were basically unsatisfied with the quality or relationships in their support networks, with the most prominent dissatisfaction among adolescents (Oslon, 2018).

Subjective well-being in children depends on their ability to be heard. In both the 10- and 12-year-old groups of participants in the Children’s Worlds study, the overall life satisfaction and the level of subjective well-being most strongly correlated with the indicator “My parents listen to me and take what I say into account.” Nevertheless, this important aspect of well-being was underrepresented in the answers: the children rated the indicator “My parents and I make decisions about my life together” the lowest (Bruk, 2019). The subjective well-being of children in children’s homes also depended heavily on their having their opinions taken into account: children who believed their views to be “completely” or “partially” considered by adults, demonstrated higher levels of well-being (Semya, 2020).

Alhough the works in this review emphasized children’s right to be heard, their subjective well-being depended on proper execution of the whole system of children’s rights, as well as a child’s subjective experience of observance or violating his/her rights. The well-being scale developed by Odinokova et al. (2018) significantly correlated (p<0.001) with answers to the question “Do you think that your rights have ever been violated at the children’s home?” At the same time, children in state care are poorly informed about their rights (Oslon, 2018), which contrasts with the results for general population, where most children know what rights they have (70.3%); in the Children’s Worlds study, Russia ranked 8th out of 34 countries on this indicator (Bruk, 2019).

The study of subjective well-being of children in state care by Oslon et al. (2018) discovered an important protective factor linked to higher levels of well-being, *i.e.,* having a personal mentor. Having a positive assessment of a relationship with a mentor increased satisfaction in all the fields that children in care viewed as problematic (support networks, future prospects, participation in decision-making), as well as adolescents’ satisfaction with living conditions in the care settings.

## Discussion

This review has shown that the approaches of Russian research teams to the development of a national CWBI are quite compatible with international practice. Although in the Children’s Worlds study Russia ranked 26th on children’s well-being, in the PISA subjective well-being domain, the proportion of Russian students who were satisfied with their lives (reported between 7 and 10 on the life-satisfaction 10-points scale), was higher than the OECD average. On the other hand, the proportion of Russian students who were dissatisfied with their lives was also higher than the OECD average. So the PISA results presented the situation with children’s subjective wellbeing in Russia as more optimistic but more polarized, compared to the results of the Children’s Worlds study. This might have related to significant variations in age groups, since the PISA focuses on 15-year-olds.

Like most of (inter)national models of children’s subjective well-being, the Russian developments toward a CWBI rely on variations of a socio-ecological approach. The structure of domains varies but is generally compatible with the UNICEF/Innocenti model. Some of the reviewed papers, along with a body of collaborative research with adolescents (*e.g.,* see Ipatova, 2020; Filippova, 2020) have indicated the importance of having an “alternative thesaurus” of CWBI, which promotes debates on subjective well-being in plain language, using terms like “happiness” and “sadness.”

The instruments used by Russian research teams included questionnaires developed specifically for CWBI purposes; standardized psychometric questionnaires (as a part of CWBI measures or as a control for various types of validity of new measures); and qualitative methods such as focus-groups, and creative and play-based tools used with small samples of children. Tools for children of various age groups and levels of abilities were available.

The development of CWBI prototypes proceeded more actively in relation to children living in state care (children’s homes); there were several comprehensive sets of tools addressed specifically to that population. Methodologically they were unusual because of their reliance on a restorative model of subjective well-being, which focuses on possibilities and resources for successful development while experiencing and overcoming adverse situations (Lent, 2004), rather than on a normative approach. Measurements were aimed at capturing the severity and dynamics of problematic behaviors and symptoms, the development of coping-strategies, and promoting access to social support and related resources, as well as changes in well-being as a core indicator of the effectiveness of social projects or reforms of the state care system.

Approbation of complex indexes for evaluation of children’s well-being in Russia has already allowed us to draw some valuable conclusions about the subjective wellbeing domain. Personal relationships with parents (or other significant adults) are of great importance for all the children, while the most problematic field of interpersonal relationships with adults and peers is school. For children in state care, the domain of interpersonal relationships was rather unsatisfactory, but having a personal mentor, who can become an attachment figure, increased their levels of well-being. This finding suggests that using both positive and negative indicators provides insights into the interplay between risk and protective factors.

Interestingly, an important role in children’s well-being belongs to the observance of children’s rights, and especially the right to be heard by adults. In the other words, the “hot spot” for subjective well-being is the intersection of close interpersonal relationships, respect, and confidence in fulfillment of one’s rights; that makes children’s participation in development of the CWBI especially important. It also exposes two types of low well-being contexts: 1) dissatisfaction with one’s social network by children in care, which might be mitigated by a mentor but cannot be completely avoided in their life situation; and 2) poor knowledge of children’s rights, a problem that can be a good target for educational interventions.

Our review was focused on approaches to subjective well-being indicators. A set of such indicators for the “Health and safety” domain was piloted by the Russian CWBI task force in 2020. Seven hundred and fifty children from ages 10 to 17 took part in in-person structured interviews in five regions (Moscow, Bashkortostan Republic, Kemerovo, Novgorod, and Nizhny Novgorod regions). Distribution of the answers to the question “*Do you have any safety concerns or experience anxiety when you go along a street in your town alone? With peers? With your close adults?*” showed that children who lived in Moscow, experienced the highest level of anxiety in the city environment (31% in Moscow vs. 26% in the whole population). Surprisingly, objective indicators picture Moscow as one of the safest Russian regions in terms of both *outcomes* (with 11 child victims of crimes per 10,000 children^1^) and *contexts* (with low incidence of violent street crimes)^2^. Such a direct juxtaposition of subjective and objective indicators reveals discrepancies between the actual situation and children’s emotional experiences, which suggests the need to test hypotheses about the underlying reasons, and to make relevant decisions in social policy.

## Conclusion

The Russian professional community has gained enough experience to develop an integral index of children’s well-being, including the subjective well-being indicators for each domain, which will allow tracking the national dynamics in the level of children’s well-being, as well as comparing it with the results obtained with analogous international indexes.

The following recommendations, drawn from our review, can inform this process:

- The Russian CWBI may be grounded in the six UNICEF/Innocenti wellbeing domains, but individual subjective well-being indicators should be adapted for Russian cultural realities.
- Maintain balance between objective and subjective indicators in the multidimensional CWBI model and search for the optimal number of items, since there are risks in making the CWBI too broad or, on the contrary, excluding some meaningful indicators, especially those difficult to measure. “Positive” and “negative”/”deficiency” indicators should also be balanced with a reasonable preference for “positive” ones.
- The balance between objective and subjective indicators can be achieved by comparing their values on the same issue, *e.g.*, police statistics of street crimes against minors vs. street (un)safety as perceived by children. Further piloting of the CWBI may prove that some objective or subjective indicators contribute little to the overall picture (*e.g.,* when subjective assessments strongly correlate with the objective data); such indicators may be removed. On the other hand, it is important to figure out subjective indicators that have no objective “proxies,” and thus make a unique contribution in our understanding of children’s well-being.
- Determine the core composition of the CWBI for longitudinal tracking in both the whole child population, as well as variations to be used with narrower target groups (*e.g.,* children in care, children with disabilities) or for cross-sectional studies to more deeply understand certain aspects of children’s well-being.
- Develop a multi-level mixed-methods model of data analysis, and supplement statistical data and large-sample questionnaires with in-depth qualitative studies using child-friendly tools (*e.g.*, storytelling- or play-based). It is also important to plan for triangulation schemes, considering both children’s and adults’ (parents’ and practitioners’) points of views.
- Children, including those from the most vulnerable groups, should be engaged in all the stages of developing and implementing the CWBI, from discussing the methodological model and indicators, to piloting measures, and collecting and interpreting the data. That will both allow making the CWBI questionnaires comprehensive and adequate for child respondents and increase the validity of obtained results.
- The roadmap for the CWBI development should embrace organizational, financial, and ethical resources and issues. On the one hand, the results of the CWBI implementation and their discussion should be made available to the public so that the relevant stakeholders could rely on them in decision-making and improvement of child and family services. To get maximum benefit from the CWBI, its implementation should be supported with an informational campaign; the results should be published in plain language and be regularly discussed by officials and expert communities (with child participation). On the other hand, the CWBI will highlight “zones of concern” in children’s well-being, which might or might not be influenced by child protection actors. It is necessary to prevent the usage of the CWBI as a tool for manipulations or punishment. To promote improvements, there should be a public system for tracking fulfillment of recommendations made on the grounds of the CWBI results.
- Since collecting comprehensive data across several domains requires lots of resources, before piloting and implementing the national CWBI, it is necessary to perform pilot research in three to five Russian regions.

## Limitations

The limitations of the current work are inherent in the scoping review approach: it relies on a relatively small number of sources that are embedded in the current national, sociocultural, and organizational contexts.

## Footnotes

1
https://tochno.st/problems/childhood

2
https://tochno.st/problems/crime

## References

[ref1] Adamson, P. (Ed.) (2007). Child Poverty in perspective: An overview of child well-being in rich countries. Report Card 7. Innocenti Research Centre. UNICEF.

[ref2] Archakova, T.O., & Garifulina, E.Sh. (2020) Measuring Children’s Subjective Well-Being in Russia: Local Practices and Federal Strategies. Monitoring of Public Opinion: Economic and Social Changes, 1, 276–295. 10.14515/monitoring.2020.1.11

[ref3] Archakova, T.O., & Garifulina, E.Sh. (2020) “Together with children”: A dialogue between children and adults during the pandemic. Comprehensive Child Studies, 2(3), 216–228. 10.33910/2687-0223-2020-2-3-216-228

[ref4] Ben-Arieh, A. (2006). Measuring and Monitoring the Well-Being of Young Children around the World. Background Paper Prepared for the Education for All Global Monitoring Report 2007 “Strong Foundations: Early Childhood Care and Education”. Paris: United Nations Educational, Scientific, and Cultural Organization. Retrieved from https://unesdoc.unesco.org/ark:/48223/pf0000147444

[ref5] Ben-Arieh, A. (2008). The child indicators movement: past, present and future. Child Indicators Research, 1, 3–16. 10.1007/s12187-007-9003-1

[ref6] Bruk, Z. (2019). Children’s Worlds National Report: Russian Federation. Retrieved from https://isciweb.org/wp-content/uploads/2020/07/Russia-National-Report-Wave-3.pdf

[ref7] Campbell, A., Converse, P.E., & Rogers, W.L. (1976). The quality of American life: Perceptions, evaluations, and satisfactions. New York. Russell Sage.

[ref8] Casas, F. (2011). Subjective social indicators and child and adolescent well-being. Child Indicators Research, 4, 4, 555–575. 10.1007/s12187-010-9093-z

[ref9] Casas, F., & Rees, G. (2015). Measures of Children’s Subjective Well-Being: Analysis of the Potential for Cross-National Comparisons. Child Indicators Research, 8, 49–69. 10.1007/s12187-014-9293-z

[ref10] Children’s Worlds Report 2020. Retrieved from https://isciweb.org/wp-content/uploads/2020/07/Summary-Comparative-Report-2020.pdf

[ref11] Cummins, R.A., & Lau, A. (2005). Manual: Personal wellbeing index — School children (3rd edn). Resource document. Melbourne, Australia: Australian Centre on Quality of Life, Deakin University. Retrieved from http://www.deakin.edu.au/research/acqol/auwbi/index-translations/wbi-school-english.pdf

[ref12] Filippova, A. (2020). O schast’e i blagopoluchii detej [On happiness and well-being in children]. Retrieved from http://deti.timchenkofoundation.org/wp-content/uploads/2020/06/О-счастье-и-благополучии-детей.pdf

[ref13] Foundation for Support of Children in Adversity. (2013). Deti v trudnoj zhiznennoj situacii: profilaktika neblagopoluchija [Children in adversity: prevention of ill-being]. Moscow. Retrieved from https://www.mintrudkchr.ru/uploadedFiles/upload-2017-07-28-14-29-34.pdf

[ref14] Heukamp, F.H., & Arino, M.A. (2011). Does Country Matter for Subjective Well-Being? Social Indicators Research, 100, 155-170. 10.1007/s11205-010-9610-y

[ref15] Huebner, E.S. (1991). Further validation of the students’ life satisfaction scale: The independence of satisfaction and affect ratings. Journal of Psychoeducational Assessment, 9, 363–368. 10.1177/073428299100900408

[ref16] Ipatova, A. (2020). Subektivnoe blagopoluchie glazami detej [Subjective well-being through children’s eyes]. Retrieved from http://deti.timchenkofoundation.org/wp-content/uploads/2020/06/A.Ипатова-благополучие-глазами-детей.pdf

[ref17] Kovacs, M. (1992). Children’s Depression Inventory (CDI). N.Y.: Multi-health Systems.

[ref18] Lee, B.J. (2014). Mapping Domains and Indicators of Children’s Well-Being. In Handbook of Child Well-Being (p. 2797). 10.1007/978-90-481-9063-8_137

[ref19] Lent, R.W. (2004). Toward a Unifying Theoretical and Practical Perspective on Wellbeing and Psychosocial Adjustment. Journal of Counseling Psychology, 51(4), 482–509. 10.1037/0022-0167.51.4.482

[ref20] Lippman, L.H., Moore, K.A., & McIntosh, H. (2011). Positive indicators of child well-being: A conceptual framework, measures, and methodological issues. Applied Research in Quality of Life, 6(4), 425–449. 10.1007/s11482-011-9138-6

[ref21] Local Practices and Federal Strategies. Monitoring of Public Opinion: Economic and Social Changes, 1, 276–295. 10.14515/monitoring.2020.1.11

[ref22] Moore, K.A., & Theokas, C. (2008). Conceptualizing a monitoring system for indicators in middle childhood. Child Indicators Research, 1, 109–128. 10.1007/s12187-008-9011-9

[ref23] Munn et al. (2018). Systematic review or scoping review? Guidance for authors when choosing between a systematic or scoping review approach. BMC Medical Research Methodology, 18, 143. 10.1186/s12874-018-0611-x 30453902 PMC6245623

[ref24] Odinokova, V.A., Rusakova, M.M., & Usacheva, N. (2017). M. Opyt ocenki blagopoluchija detej v uchrezhdenijah dlja detej-sirot [Experience of evaluation of well-being of children living in state care institutions for orphans]. Monitoring obshhestvennogo mnenija: Jekonomicheskie i social’nye peremeny [Monitoring of Public Opinion: Economic and Social Changes], 2, 129–144. https://doi:10.14515/monitoring.2017.2.08

[ref25] Osin, E.N., & Rasskazova, E.I. (2013). Kratkaja versija testa zhiznestojkosti: psihometricheskie harakteristiki i primenenie v organizacionnom kontekste [A short version of the hardiness test: Psychometric characteristics and application in an organizational context]. Vestnik Moskovskogo universiteta. Serija 14. Psihologija [Moscow University Psychology Bulletin. Series 14. Psychology], 2, 147–165.

[ref26] Oslon, V.N. (2019). Monitoring subektivnogo blagopoluchija vospitannikov kak instrument ocenki kachestva dejatelnosti organizacij dlja detej-sirot [The monitoring of subjective well-being of children in children’s homes as a tool for evaluation of the quality of services in state care]. In T.A. Basilova, E.G. Dozorceva, T.A. Egorenko … L.I. Jelkoninova, (Eds.), VII Vserossijskaja nauchno-prakticheskaja konferencija po psihologii razvitija (Chtenija pamjati L.F. Obuhovoj) “Vozmozhnosti i riski cifrovoj sredy”. Sbornik materialov konferencii (tezisov). Tom 2. [The VIIth Russian applied-scientific conference on developmental psychology. Vol. 2] (pp. 197–201). Moscow: MSUPE.

[ref27] Oslon, V.N., Semya, G.V., Prokopeva, L.M., & Kolesnikova, U.V. (2020). Operacional’naja model’ i instrumentarij dlja izuchenija sub#ektivnogo blagopoluchija detej-sirot i detej, ostavshihsja bez popechenija roditelej [Operational Model and Tools for Studying Subjective Well-Being of Orphans and Children Without Parental Care]. Psikhologicheskaya nauka i obrazovanie [Psychological Science and Education], 25(6), 41–50. https://doi:10.17759/pse.2020250604

[ref28] PISA 2018: Draft analytical frameworks. OECD, 2016. Retrieved from https://www.oecd.org/pisa/data/PISA-2018-draft -frameworks.pdf

[ref29] PISA 2018: Insights and Interpretations. Retrieved from https://www.oecd.org/pisa/PISA%202018%20Insights%20and%20Interpretations%20FINAL%20PDF.pdf

[ref30] Proekt Plana osnovnyh meroprijatij, provodimyh v ramkah Desjatiletija detstva, na period do 2027 goda [Prospect plan for the main activities in the frames of “Childhood Decade” up to 2027]. Retrieved from https://www.oprf.ru/about/structure/structurenews/newsitem/54526

[ref31] Rees, G., Savahl, S., Lee, B.J., & Casas, F. (Eds.). (2020). Children’s views on their lives and well-being in 35 countries: A report on the Children’s Worlds project, 2016-19. Jerusalem, Israel: Children’s Worlds Project (ISCWeB). Retrieved from https://isciweb.org/wp-content/uploads/2020/07/Childrens-Worlds-Comparative-Report2020.pdf

[ref32] Rogozin, D., Ipatova, A., & Garigulina, E. (2019). Detskoe (ne)blagopoluchie poisk parametrov izmerenija [Children’s well/ill-being: in search for measurement indicators]. Retrieved from http://deti.timchenkofoundation.org/wp-content/uploads/2020/01/мецкий-благословите-Д-Рогозин-A.Ипатова.pdf

[ref33] M. (1965). Society and the adolescent self-image. Princeton, NJ: Princeton University Press. 10.1515/9781400876136

[ref34] Russell, J.A. (2003). Core affect and the psychological construction of emotion. Psychological Review, 110(1), 145–172. 10.1037/0033-295X.110.1.145 12529060

[ref35] Sandin, B. (2013). History of Children’s Well-Being. In A. Ben-Arieh, F. Casas, I. Frønes, & Korbin, J.E. (Eds.), Handbook of Child Well-Being: Theory, Indicators, Measures and Policies (pp. 31–86). Dordrecht: Springer.

[ref36] Semya, G.V. (2020). Subektivnoe blagopoluchie glazami detej [Subjective well-being through children’s eyes]. Retrieved from http://deti.timchenkofoundation.org/wp-content/uploads/2020/06/E.+1A0F-+83G1?/2=D61-34&567648.21.pdf

[ref37] Seligson, J.L., Huebner, E.S., & Valois, R.F. (2003). Preliminary validation of the Brief Multidimensional Students’ Life Satisfaction Scale (BMSLSS). Social Indicators Research, 61(2), 121–145. 10.1023/A:1021326822957

[ref38] Schur, V.G. (1982). Metodika “Lesenka” [Self-Assessment Ladder]. In Psihologija lichnosti: teorija i jeksperiment [Personality psychology: theory and experiments]. Moscow.

